# Diagnostic Yield and Cost-Effectiveness of “Dynamic” Exome Analysis in Epilepsy with Neurodevelopmental Disorders: A Tertiary-Center Experience in Northern Italy

**DOI:** 10.3390/diagnostics11060948

**Published:** 2021-05-25

**Authors:** Costanza Varesio, Simone Gana, Alessia Asaro, Elena Ballante, Raffaella Fiamma Cabini, Elena Tartara, Michela Bagnaschi, Ludovica Pasca, Marialuisa Valente, Simona Orcesi, Cristina Cereda, Pierangelo Veggiotti, Renato Borgatti, Enza Maria Valente, Valentina De Giorgis

**Affiliations:** 1 Department of Child Neurology and Psychiatry, IRCCS Mondino Foundation, 27100 Pavia, Italy; ludovica.pasca01@universitadipavia.it (L.P.); simona.orcesi@mondino.it (S.O.); renato.borgatti@mondino.it (R.B.); valentina.degiorgis@mondino.it (V.D.G.); 2 Department of Brain and Behavioral Sciences, University of Pavia, 27100 Pavia, Italy; michela.bagnaschi01@universitadipavia.it; 3 Medical Genetics Unit, IRCCS Mondino Foundation, 27100 Pavia, Italy; simone.gana@mondino.it (S.G.); alessia.asaro@mondino.it (A.A.); marialuisa.valente@asl.taranto.it (M.V.); enzamaria.valente@unipv.it (E.M.V.); 4 BioData Science Center, IRCCS Mondino Foundation, 27100 Pavia, Italy; elena.ballante01@universitadipavia.it; 5 Department of Mathematics, University of Pavia, 27100 Pavia, Italy; raffaellafiamma.cabini01@universitadipavia.it; 6 Istituto Nazionale di Fisica Nucleare Section of Pavia, 27100 Pavia, Italy; 7 Epilepsy Center, IRCCS Mondino Foundation, 27100 Pavia, Italy; elena.tartara@mondino.it; 8 Laboratory of Clinical Pathology Microbiology and Genetics, SS. Annunziata, 74100 Taranto, Italy; 9 Molecular Genetics and Cytogenetics Section, IRCCS Mondino Foundation, 27100 Pavia, Italy; cristina.cereda@mondino.it; 10 Genomic and Post-Genomic Unit, IRCCS Mondino Foundation, 27100 Pavia, Italy; 11 Pediatric Neurology Unit, Vittore Buzzi Hospital, 20100 Milano, Italy; pierangelo.veggiotti@unimi.it; 12 Biomedical and Clinical Sciences Department, Luigi Sacco Hospital, University of Milan, 20100 Milano, Italy; 13 Department of Molecular Medicine, University of Pavia, 27100 Pavia, Italy

**Keywords:** epilepsy, neurodevelopmental disorders, developmental epileptic encephalopathies, next-generation sequencing techniques, exome sequencing, virtual genetic panels, dynamic approach

## Abstract

Background: The advent of next-generation sequencing (NGS) techniques in clinical practice led to a significant advance in gene discovery. We aimed to describe diagnostic yields of a “dynamic” exome-based approach in a cohort of patients with epilepsy associated with neurodevelopmental disorders. Methods: We conducted a retrospective, observational study on 72 probands. All patients underwent a first diagnostic level of a 135 gene panel, a second of 297 genes for inconclusive cases, and finally, a whole-exome sequencing for negative cases. Diagnostic yields at each step and cost-effectiveness were the objects of statistical analysis. Results: Overall diagnostic yield in our cohort was 37.5%: 29% of diagnoses derived from the first step analysis, 5.5% from the second step, and 3% from the third. A significant difference emerged between the three diagnostic steps (*p* < 0.01), between the first and second (*p* = 0.001), and the first and third (*p* << 0.001). The cost-effectiveness plane indicated that our exome-based “dynamic” approach was better in terms of cost savings and higher diagnostic rate. Conclusions: Our findings suggested that “dynamic” NGS techniques applied to well-phenotyped individuals can save both time and resources. In patients with unexplained epilepsy comorbid with NDDs, our approach might maximize the number of diagnoses achieved.

## 1. Introduction

Childhood-onset epilepsies are known to be associated with various degrees of neurodevelopmental disorders (NDDs) in approximately 25% of cases [1]. Many of these syndromes persist into adulthood, although their prevalence is still difficult to estimate [2,3]. The concomitant presence of epilepsy and NDDs is due to at least two mechanisms, not mutually exclusive. The first one is linked to the detrimental effect of uncontrolled epileptic seizures and of epileptiform activities on cortical networks [4], and the second one may be linked to a single defect, in particular of genetic origin, sufficient to generate, on the one hand, epileptic seizures, and on the other hand, global developmental delay and/or neurocognitive comorbidity, irrespective of an underlying structural pathology [5,6]. As the two mechanisms may play a role in the same individual and a single genetic defect can act through both mechanisms, the term developmental and epileptic encephalopathy (DEE) has been coined to refer to conditions in which epilepsy and NDDs coexist based on these two pathogenic mechanisms [6,7]. The genetic landscape of epilepsy with NDDs and DEE is broad and complex [8]. For this reason, there are many options for diagnostic investigations on this topic [9]. The genetic investigation applied in the first instance is represented by the array comparative genome hybridization (a-CGH) to detect copy number variants [9]. In the last decades, progress in technology development and affordability of next-generation sequencing techniques (NGS) (including gene panels, clinical exome sequencing, whole-exome sequencing, and whole-genome sequencing) led to a significant advance in gene discovery, with several genes implicated in epilepsy with NDD [10].

The reported diagnostic yield of NGS techniques in patients with epilepsy varies considerably (from 10% to 84.6%, comparable between genetic panels and WES) [11,12].

Along with the advantages linked to the rapid identification of causative genes, the application of NGS in clinical practice presents some challenges, mainly represented by variant interpretation and clinical correlations [11]. In 2013, Neveling and colleagues [13] observed that genetic causes of heterogeneous diseases, including neurological disorders, could be detected by exome sequencing followed by targeted analysis of disease-specific gene sets, thus optimizing the interpretation of the results and minimizing the risk of incidental findings. In line with this assessment, a recent review by Pellacani and colleagues [12] suggests that customized gene panels for epilepsy may be of great diagnostic value when they are rationally selected and applied in carefully phenotyped patients. They also stated that, in the same vein, “ideal” panels could also be useful when performing whole-exome sequencing or whole-genome sequencing to prioritize analysis.

To date, few studies have focused attention on health-economic evidence of NGS techniques in clinical settings. In a relatively recent review by Schwarze and colleagues [14], the authors concluded that the current health-economic evidence base to support the more widespread use of exome sequencing and genome sequencing in clinical practice is limited, and studies focused on careful evaluation of the cost-effectiveness of these tests in clinical practice are urgently needed.

Herein, we aimed to describe the diagnostic efficiency of a proband-only dynamic NGS approach, namely, to evaluate the diagnostic yield and assess the value of reinterpreting genetic test results in a cohort of patients with epilepsy associated with NDDs and/or DEEs, and discuss the advantages, disadvantages, and cost-effectiveness of our diagnostic strategy.

## 2. Materials and Methods

### 2.1. Patients and Phenotyping

We conducted a retrospective, observational study that involved 72 consecutive patients presenting epilepsy and NDDs or DEE, recruited over the past three years (2017–2020) in a tertiary northern Italy Neurological Institute, according to the Declaration of Helsinki. The ethics committee of our Institute approved the protocol (P-20200099763).

All patients met the following inclusion criteria: (1) presence of epilepsy associated with neurodevelopmental disorder or DEE, (2) referral to our Center for neurological and genetic investigations, (3) no causative abnormalities detected by previous metabolic, infectious, immunologic, or genetic workup (including karyotype, array-CGH, or targeted Sanger sequencing), and (4) absence of magnetic resonance imaging (MRI) findings suggestive of a structural etiology. Patients with normal cognitive functions were excluded.

NDDs were determined based on DSM5 definitions [15]. Epilepsy characterization was determined according to the International League Against Epilepsy classification [16].

Phenotypic data, including seizure and epilepsy characterization, electroencephalograms, brain imaging reports, metabolic studies, developmental history, and previous genetic testing reports, were reviewed by a team of pediatric and adult epileptologists and genetic counselors with expertise in epilepsy and variant interpretation.

### 2.2. Genetic Workup

After obtaining written informed consent for genetic testing from patients’ parents or legal guardians, proband-only clinical exome sequencing (CES), using the SureSelect QXT Target Enrichment System (Agilent Technologies, Santa Clara, CA) on a NextSeq500 platform (Illumina, San Diego, CA), was performed according to the manufacturers’ protocols between 2017 and June 2019. Then, based on technical laboratory implementation, a whole-exome sequencing (WES), using the Twist Human Core Exome Kit (Twist Bioscience HQ, South San Francisco, CA) on a NovasSeq6000 platform (Illumina, San Diego, CA), was performed according to the manufacturers’ protocols since July 2019. SNVs and InDels variants were filtered according to the usual workflows considering their frequency in the general population, the indication of their disease-causing potential by in silico prediction tools, and segregation analysis in family members [17].

The variants were classified using the American College of Medical Genetics (ACMG) criteria [18], and those deemed likely or definitely pathogenic, both considered in the calculation of detection rate, were confirmed using Sanger sequencing. Sanger sequencing was also performed to confirm variants in parents.

The approach adopted to select “virtual gene panels” included genes recurrently mutated in DEE and genes shared among epilepsy and NDDs networks. In line with what was proposed by Snoeijen-Schouenaars and colleagues [19], in the first step, based on the strength of evidence existing in the literature at the time of the method implementation in clinical practice, only variants in known DEE and epilepsy with NDDs were considered by using a filter for a specific panel (first designed virtual panel of 135 genes—complete list in Additional file: Supplementary Table S1). Based on a more recent literature review (carried out in the first quarter of 2020), we designed a second “virtual panel” of genes related to new syndromes encompassing DEE and epilepsy with NDDs reported in more recent studies (second step analysis adding 84 genes for the CES and 162 genes for the WES group—complete list in Additional file: Supplementary Table S1). Patients with negative results at the first step were then retrospectively reanalyzed. If both the “virtual panel” analyses revealed non-diagnostic results, our “dynamic approach” allowed to extend analysis to the total exhaustive CES and WES data (third step) to identify extremely rare variants or new disease genes.

### 2.3. Costs

In the event of a suspected genetic syndrome, the Regional health system in Lombardia, Italy, provides a reimbursement of 2072.74 euros for each genetic analysis conducted using NGS techniques. In this scenario, we defined the NGS diagnostic pathway’s cost as the public reimbursement cost for NGS analysis provided by our local socialized health system.

### 2.4. Plan of Analysis

We estimated the diagnostic yields and cost per diagnosis for each diagnostic pathway following the approach described by Stark and colleagues [20] and Palmer and colleagues [21].

We assumed that patients who received a diagnosis through the standard diagnostic pathway (first- and second-step assessments and then complete NGS data analysis as the final diagnostic step) would also have received it by having WES as their first-level test.

Diagnostic yields at each step of the standard diagnostic pathway were compared through Cochran’s Q test for the comparison of proportion in paired samples, and post hoc pairwise comparisons are performed using McNemar’s test.

The statistical significance level was defined with *p* ≤ 0.05.

We compared the incremental cost per additional diagnosis for the WES approach as a first-step test and as a third-step test over the standard diagnostic pathway (first-step assessment and then second-step assessment). We used a bootstrapping simulation to assess the uncertainty associated with each diagnostic result and to generalize results obtained on the specific sample enrolled. Using this technique, we created 500 replicated datasets and evaluated the additional cost and the additional number of diagnoses for each of these datasets, thus generating 500 estimates of each outcome. Results of the bootstrapping simulation are represented in the cost-effectiveness plane.

All statistical analyses were conducted in R 4.0.2, RStudio (R Core Team, 2020; R Foundation for Statistical Computing, Vienna, Austria; https://www.R-project.org/ 15 march 2021), and figures were produced using the package ggplot2 (H. Wickham; Springer-Verlag New York, 2016).

## 3. Results

### 3.1. Demographics and Phenotype

In this retrospective study, 72 unrelated patients with unexplained epilepsy and comorbid NDDs or DEE were included. They underwent genetic workup between 2017 and 2020.

### 3.2. Molecular Diagnostic Yield of the Cohort

Under the reasonable assumption that patients who received a diagnosis in the previous step would also have received diagnosis in the following steps, the diagnostic yields were evaluated on the total court of 72 subjects not only at the first step, but also at the second and at the third steps, instead of the restricted sample of non-diagnosed subjects.

Our cohort’s overall yield of pathogenic or likely pathogenic variants was 37.5% (27/72 patients). In about a quarter of cases, the genetic diagnosis was reached in adulthood. We investigated whether diagnostic yield varied between different steps. The diagnostic rate increased after the following steps, and 29% of patients (21/72) obtained a genetic diagnosis at the first level and therefore required no further analysis.

The exome-based analysis approach enabled us to reanalyze patients with negative or inconclusive results at the first level. Reanalysis provided an additional 5.5% of diagnosis (4/72) at the second level, whereas an additional 3% (2/72) derived from the third level.

**Figure 1:**
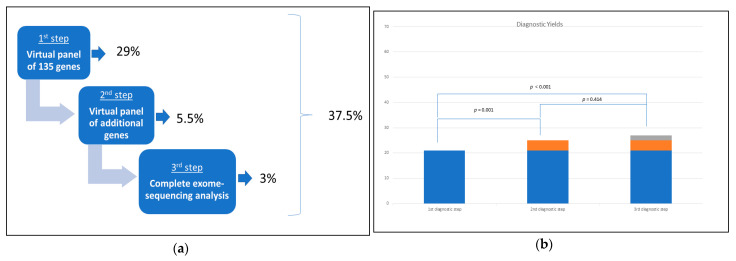
(**a**) Diagnostic yields increase in the three genetic-workup steps with the statistically significant difference between the three different steps. (**b**) 21 out of 72 patients (29%) obtained a genetic diagnosis at the first “virtual panel” of 135 genes. The exome-based reanalysis of additional genes more recently associated to epilepsy and NDDs and/or DEEs provided a genetic diagnosis in 4 patients (5.5%), whereas another 2 patients were genetically diagnosed exploring the complete CES/WES data (3%).

Comparing the diagnostic yield of the three genetic-workup levels, a statistically significant difference between the three diagnostic levels emerged (*p* < 0.01) (Figure 1a,b). In particular, from the comparison of diagnostic levels by pair, a strong statistically significant difference was observed between the first and the second (*p* = 0.001) and between the first and the third (*p* < 0.001), while the comparison between the second and the third was not significant (*p* = 0.414).

Clinical, epileptological, and genetic characteristics of positive cases are provided and fully detailed in Table 1.

Detection of pathogenic or likely pathogenic variants was overall obtained in 27 out of 72 patients (37.5%).

### 3.3. Cost Analysis

Cost analysis was performed based on the regional reimbursement of 2072.74 euros provided for each NGS technique applied per patient, independently from the different techniques.

We compared two different models to the standard diagnostic protocol (first and second step). Model 1 is defined as the two-step panels and the exome sequencing as the third-step test, and Model 2 is the exome sequencing as a first-step test.

In the first model, represented by a multistep analysis (consecutive two panels and exome sequencing), we revealed an estimated cost per diagnosis of 13,050.59 euros for the medical system. Alternatively, the exome sequencing since the beginning with the analysis and reanalysis through “dynamic panels” (second model) proved to be a cost-saving pathway, as it determined an expense for the health system of 5527.307 euros per diagnosis. As a reference model, a two-step panels approach was considered, with an average cost per diagnosis of 10,197.88.

**Figure 2:**
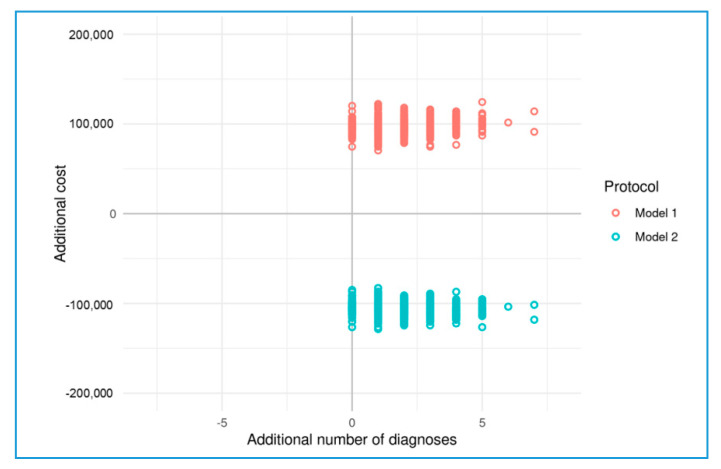
Cost-effectiveness plane comparing two different models to the standard diagnostic protocol (first and second step). Model 1: exome sequencing as the third-step test, and Model 2: exome sequencing as the first-step test. The additional number of diagnoses (effect) is plotted on the x-axis and the additional costs on the y-axis. Each point in the plane is derived from one of the 500 bootstrapped simulations and is represented on four quadrants. Since all points are distributed in the two right quadrants, both models produce a higher (or equal) number of diagnoses than the reference protocol. The area below the x-axis is cost saving, while the area above is cost increasing. This indicates that exome sequencing from the beginning with stepwise “virtual panel” testing is both clinically superior and cost-effective compared to the standard care and it is referred to as an economically “leading” strategy.

The cost-effectiveness plane (Figure 2) indicates that our exome sequencing approach since the beginning was leading in terms of cost savings and higher diagnostic rate, compared to a multi-step panel approach with exome sequencing as the last tier.

## 4. Discussion

In recent years, implementation of rapid transformations and techniques have led to incorporating NGS techniques in clinical practice, keeping advantages and limitations in diagnostic yields, costs, ease of analysis, and detectable genetic variant types. Previous studies have demonstrated that the NGS approach is an efficient tool for the diagnosis of epileptic disorders, in particular in DEE and in those conditions where epilepsy is comorbid with neurodevelopmental disorders [11,12,22].

Currently, there is no consensus regarding the most appropriate NGS approach to patients with unexplained epilepsy and comorbid NDDs in the clinical field: whether to apply a customized genetic panel or a WES as the first diagnostic level remains a matter of debate [11,12,23].

In this study, we reported results of a “dynamic” exome-based analysis in terms of diagnostic yield and costs per diagnosis in a clinical setting of a tertiary Neurological Institute in Italy with both pediatric and adult epilepsy centers. Results were considered in terms of comparison of diagnostic yield and cost-effectiveness of diagnostic approaches. Our results should be interpreted under the assumption that the genetic diagnosis provided through our exome-based dynamic approach would have also been made through a multistep NGS gene panels approach with exome sequencing performed as the last step. In our clinical practice, a diagnostic “virtual” panel of 135 genes (first step) with clear evidence for causing epilepsy with NDDs or DEE was designed in conjunction with clinical, molecular geneticists, and neurologists. It was later expanded to include other newly discovered genes or genes less frequently related to epilepsy (second step), allowing retrospective reanalysis of inconclusive data. Eventually, in negative cases, the analysis was extended to all genes associated with any known mendelian disorder (third step).

The overall diagnostic yield of 37.5% in our sample was in line with a previous study on pediatric epilepsies associated with NDDs and DEE [11,24,25], thus suggesting that proband-only exome sequencing techniques are helpful diagnostic tools for these patients in a clinical setting [11,26,27]. Such diagnostic yields appeared to be higher than those observed in patients with “idiopathic” epilepsy, indicating that the application of NGS techniques depicts a higher clinical value in individuals with epilepsy and NDDs or DEE [11].

Considering diagnostic yield for each level, it appeared to be 29% for the first step, an additional 5.5% was observed for the second step reanalysis, and a further 3% for the third step. Although more than two-thirds of diagnoses were achieved at the first analysis, we demonstrated that a significantly higher genetic diagnosis was warranted by second and third reanalysis. Notably, in our sample, a statistically significant difference was observed in the number of diagnoses obtained between the first and the second level and between the first and the third.

Our data highlighted the importance of iterative reanalysis in patients with negative results at the first diagnostic approach. The reinterpretation of negative or inconclusive genetic data has proven to be an effective means of revealing new disease-causing variants [27,28,29]. As also demonstrated by our results, it can significantly increase diagnostic yield. Since our approach is “virtual,” it enables flexible management over time, permitting us to re-evaluate the raw data several times and update the list of analyzed genes, incorporating the newly discovered ones. From this perspective, as proposed by the American College of Medical Genetics and Genomics (ACMG) [18,30], we recommend all undiagnosed cases be re-analyzed at a minimum frequency of every two years, including more genes recently identified as disease-causing candidates.

Achieving a genetic diagnosis is crucial for families. It provides relief from uncertainty, it puts an end to “diagnostic odyssey” by avoiding additional unnecessary investigations, and it may guide therapeutic choices. Physicians should clearly explain to patients that medical knowledge is continually evolving, and genetic test interpretation might change over time; thus, negative results today do not mean the patients do not have a specific genetic etiology, which will be identified over time. The genetic reanalysis allowed to better understand and also expand the phenotypic characterization of some gene variants causing epilepsy with neurodevelopmental disorders, also in adult patients, in whom it was possible to retrospectively evaluate the evolution of the disease over time.

Although the topic is outside our work scope, we believe some ethical issues deserve some considerations. One key difference between the three levels of analysis is represented by the number of variants identified. The first and the second “virtual panels” are phenotype-driven, meaning that the analyzer could only focus on variants in phenotype-related genes, automatically filtering genes unrelated to patients’ symptoms. The passage to the complete data analysis, along with the better chance of identifying pathogenetic variants, significantly enlarges the likelihood of incidental or unsolicited findings [31]. The management of this eventuality should be taken into consideration given a progressive implementation of the use of exome in clinical practice.

Although the cost-effectiveness of WES application in clinical settings is still debated [23,32], an economic evaluation is crucial for its implementation to appropriately improve diagnostic rates. Our data confirm previous observations by Stark and colleagues [20] that applying WES at the last diagnostic trajectory resource is not a cost-effective option. Indeed, the gene panels step process would determine an incremental cost for additional diagnosis more than twice as high as the application of CES/WES, analyzed stepwise through dynamic panels, from the beginning.

A limitation of our study might be represented by our proband-only approach. This approach has proven effective in uncovering etiology of DEEs or epilepsy with NDDs in a routine clinical practice [11,25]. In a research context, a trio approach might be more effective in discovering new disease-associated genes and in increasing confidence on the role of variants of unknown significance. However, in a clinical setting, as our context is, management of trio testing would not be feasible due to unsustainable costs for parental exome-sequencing and possible unavailability of parental DNA. As proposed by Balciuniene and colleagues [27], we promote the hypothesis that a possible strategy to overcome this limitation could be to obtain parents’ samples (when available) simultaneously with the proband and to perform parental testing to support interpretation of variants identified in the proband.

## 5. Conclusions

Currently, no practice guidelines are focusing on the best approach to clinical genetic testing in individuals with epilepsy. Overall, our findings suggested that “dynamic” NGS techniques applied to appropriate well-phenotyped individuals and interpreted by a multidisciplinary team can save both time and resources. Indeed, a multidisciplinary team (including epileptologists, genetic counselors, and biologists with expertise in epilepsy genetics and variant interpretations) and a “dynamic” approach to all individuals with unexplained epilepsy comorbid with NDDs or DEE might maximize the number of patients for whom a genetic diagnosis could be achieved.

## Figures and Tables

**Table 1:** Clinical, epileptological, and genetic characteristics of probands with pathogenic or likely pathogenic variants grouped based on diagnostic level. Abbreviations: ASD, autism spectrum disorder; DEE, developmental and epileptic encephalopathy; DS, Dravet Syndrome; LGS, Lennox-Gastaut Syndrome; LKS, Landau-Kleffner Syndrome; F, female; ID, intellectual disability; M, male; m, months; NDD, neurodevelopmental disorder; Y, years.

Proband	Gender	Age	Age at Diagnosis	Gene	Coding Variants	Transcript	Genotype	Protein Variants	Epileptic Diagnosis	NDD Diagnosis
	**First Diagnostic Level**									
#1	M	17 y	15y	***GRIN2B***	c.2444_2446delTTG	NM_000834.5	Het	p.Ile815_Asp816delinsAsn	DEE	Rett-like phenotype
#2	F	41 y	40y	***IQSEC2***	c.848delG	NM_001111125.3	Het	p.Gly283AlafsTer23	DEE	Severe ID
#3	F	7 y	4 y	***MECP2***	c.808C>T	NM_004992.4	Het	p.Arg270Ter	DEE	Rett Syndrome
#4	F	20 y	19 y	***GNAO1***	c.674G>A	NM_138736.3	Het	p.Cys225Tyr	DEE	Profound ID
#5	M	32 y	29y	***CHD2***	c.3951G>T	NM_001271.4	Het	p.Leu1317Phe	FE	Severe ID, ASD
#6	F	13 y	12 y	***PURA***	c.10C>T	NM_005859.5	Het	p.Arg4Ter	DEE	Profound ID
#7	M	6 y	5 y	***KCNQ2***	c.502T>A	NM_172107.4	Het	p.Phe168Ile	GE	Profound ID
#8	M	13 y	9 y	***KCNQ2***	c.365C>T	NM_172107.4	Het	p.Ser122Leu	GE	Profound ID, ASD
#9	F	14 y	12 y	***IQSEC2***	c.2201_2202delAC	NM_001111125.3	Het	p.Tyr734SerfsTer7	FE	ID
#10	M	4 y	6 m	***CDKL5***	c.601_603delCTT	NM_003159.2	Hemi	p.Leu201del	DEE	Rett-like phenotype
#11	M	5 y	1 y	***CDKL5***	c.569delA	NM_003159.2	Hemi	p.Lys190SerfsTer38	DEE	Rett-like phenotype
#12	F	29 y	28 y	***SLC2A1***	c.988C>T	NM_006516.4	Het	p.Arg330Ter	FE	Moderate ID
#13	M	32 y	28 y	***ADSL***	c.1277G>A	NM_000026.4	Hom	p.Phe426His	DEE	Profound ID
#14	M	7 y	4 y	***SCN1A***	c.3610T>C	NM_001202435.3	Het	p.Trp1204Arg	DEE (DS)	Psychomotor delay
#15	F	9 y	6 y	***KCNT1***	c.2627A>G	NM_020822.3	Het	p.Tyr876Cys	FE	Moderate ID
#16	F	7 y	3 y	***SCN8A***	c.5630A>G	NM_014191.4	Het	p.Asn1877Ser	FE	Moderate ID, autistic-like features
#17	F	12 y	9 y	***SLC2A1***	c.940G>A	NM_006516.4	Het	p.Gly314Ser	GE	Mild ID
#18	M	8 y	7 y	***GRIN2A***	c.2189A>G	NM_000833.5	Het	p.Tyr730Cys	DEE (LKS)	Moderate ID
#19	M	17 y	16 y	***IQSEC2***	c.1075C>T	NM_001111125.3	Hemi	p.Arg359Cys	GE	Moderate ID
#20	M	6 y	3 y	***GABRB3***	c.39G>A	NM_021912.5	Het	p.Trp13Ter	GE	Psychomotor delay
#21	F	7 y	6 y	***SLC6A1***	c.1229A>C	NM_003042.4	Het	p.Asp410Ala	GE	Mild ID
	**Second Diagnostic Level**									
#22	F	21 y	20 y	***DYNC1H1***	c.12161_12162delATins TGGTTATGATGCCA	NM_001376.5	Het	p.His4054delinsLeuVal MetMetPro	GE	Mild ID
#23	F	11 y	10 y	***PIGN***	c.1434+5G>A	NM_176787.5	Hom	-	DEE	Profound ID
#24	F	4 y	3 y	***PIGN***	c.1251+1G>T	NM_176787.5	Het	-	DEE	Profound ID
				***PIGN***	c.2399G>A	NM_176787.5	Het	p.Gly800Glu		
#25	F	22 y	21 y	***GNB1***	c.233A>G	NM_002074.5	Het	p.Lys78Arg	DEE	Profound ID
	**Third Diagnostic Level**									
#26	M	16 y	15 y	***PRRT2***	c.649dupC	NM_001256442.2	Het	p.Arg217ProfsTer8	Focal epilepsy	Mild ID
#27	F	6 y	5 y	***PIGC***	c.859G>T	NM_153747.2	Hom	p.Glu287Ter	DEE	Profound ID

## Data Availability

The data that support the findings of this study are available from the corresponding author, C.V., upon reasonable request.

## Supplementary Materials

The following are available online at https://www.mdpi.com/article/10.3390/diagnostics11060948/s1: Supplementary Table S1.
